# A bibliometric and visual analysis of colorectal cancer-diabetes comorbidity

**DOI:** 10.1007/s12672-026-04447-w

**Published:** 2026-01-22

**Authors:** Junyu Long, Xuewei Shi, Yandong Huang, Qingyang Bai, Kai Feng

**Affiliations:** 1 Baotou Medical College, Inner Mongolia University of Science and Technology, Inner Mongolia, China; 2 Tumor Center Department, The First Affiliated Hospital of Baotou Medical College, Inner Mongolia University of Science and Technology, Inner Mongolia, China

**Keywords:** Colorectal cancer, Diabetes, Bibliometrics, Visual analysis

## Abstract

**Background:**

Colorectal cancer (CRC), a leading cause of global cancer-related mortality, exhibits a complex bidirectional relationship with diabetes mellitus (DM). Epidemiological evidence indicates that DM significantly increases the risk of CRC, while CRC progression may exacerbate diabetic complications; however, the underlying mechanisms remain incompletely understood. This study aimed to conduct a bibliometric analysis of the literature on COLORECTAL CANCER-DIABETES comorbidity to identify research trends, collaborative networks, and emerging thematic foci.

**Methods:**

We performed a comprehensive search in the Web of Science Core Collection (WoSCC) database to retrieve relevant literature. Using analytical and visualization tools, including CiteSpace and VOSviewer, we examined publication trends, major contributing countries and institutions, collaboration networks, and keyword evolution to map the current research landscape in this field.

**Result:**

Annual publication output on COLORECTAL CANCER-DIABETES comorbidity increased markedly after 2005, reflecting growing recognition of the metabolic-cancer interplay and expanded research funding. The United States and China were the dominant contributors, with Harvard University and Birmingham Women’s Hospital among the most productive institutions. Keyword analysis revealed emerging research clusters such as *“diabetes-associated centrosome amplification*,*” “metformin’s therapeutic potential*,*”* and *“anastomotic leakage.”*

**Conclusion:**

Research on the interplay between colorectal cancer and diabetes is gaining increasing priority. Future efforts should focus on elucidating molecular mechanisms—such as oxidative stress and angiogenesis dysregulation—refining epidemiological models, and translating findings into clinical practice. Developing precision medicine strategies for CRC screening and treatment in diabetic populations, alongside the exploration of novel biomarkers and therapeutic targets, will be essential to improve patient prognosis.

**Supplementary Information:**

The online version contains supplementary material available at 10.1007/s12672-026-04447-w.

## Introduction

Diabetes mellitus (DM) and colorectal cancer (CRC) are both diseases with high treatment costs that place a heavy financial burden on individuals, society, and the nation [1, 2]. Diabetes is a common chronic disease in the population, mainly characterised by insulin secretion disorders, insulin utilisation disorders or a combination of both deficiencies, and is mainly classified into type 1 diabetes, type 2 diabetes mellitus (T2DM) and gestational diabetes, with T2DM accounting for 90% of the total number of people suffering from the disease [3]. Worldwide, diabetes is one of the leading causes of death among adults, and its prevalence and incidence continue to rise. The 9th edition of the International Diabetes Federation (IDF) Atlas shows that the number of people with diabetes is expected to increase to 700 million globally by 2045 [4]. Most CRC cases evolve from adenomatous polyps [5]. Previous studies have suggested that adenomatous polyps may evolve as neoplastic precursor lesions from cancer stem cells or stem cell-like cells located at the base of the colonic crypts [6]. CRC was estimated to be the third most common cause of morbidity (9.6 per cent) and the second most common cause of mortality (9.3 per cent) globally in 2022 [7]. The 8th edition of the American Joint Committee on Cancer (AJCC) cancer staging manual suggests that the relative survival rate for patients with stage IV CRC is only about 40% [8]. Previous EUROCARE studies on cancer survival analysis in European countries have shown that CRC patients aged 60–69 had an average five-year cumulative relative survival rate of 40% and that the prognosis becomes poorer with increasing age at diagnosis [9].

A complex bidirectional relationship exists between diabetes and CRC. Diabetes is one of the risk factors for CRC, and CRC may also contribute to the progression of diabetes. Most diabetic patients are associated with an increased incidence of various solid tumours, such as pancreatic cancer, breast cancer, cancers of the hepatobiliary system and CRC. There is a positive correlation between the risk of CRC in patients with diabetes compared to those without diabetes [10, 11]. In addition, studies have shown that CRC patients have a significantly higher risk of developing DM within 5 years of diagnosis compared to non-CRC patients [12]. Shared risk factors for both disorders include insulin resistance (IR), hyperglycemia, and hyperinsulinemia [11, 13, 14]. Prolonged hyperglycemia can seriously damage the function of human organs, and the worse the daily glycaemic control, the more serious the damage to the target organs [15].

Currently, there is limited bibliometric research output focusing on CRC from the perspective of diabetes, with a notable lack of targeted studies in this area, which warrants further investigation. In 1969, Alan Pritchard defined bibliometrics as a discipline that employs mathematical and statistical methods to analyse scholarly publications [16]. As an emerging discipline with extensive application potential, particularly in the field of medical science, the use of CiteSpace enables quantitative analysis of research progress in target domains, thereby providing robust data support for future studies and policy formulation.

This study conducted a bibliometric analysis of research outputs on colorectal cancer-diabetes comorbidity from 1990 to 2024, identified key research hotspots in the field, and provided novel perspectives for subsequent investigations.

## Materials and methods

### Data sources

In the literature search, initially, we selected one of the three major databases, the Web of Science Core Collection (WoSCC), Scopus and Google Scholar, which are the main sources of citation data. Google Scholar provides richer citation results among the three, but its data sources are not all specialised academic journals. Scopus is a newcomer in the field of bibliometric research, but its data content is relatively new. Although WoSCC is not perfect, it is still regarded as the gold standard in the field of bibliometrics [16]. Meanwhile, the data analysed by CiteSpace is based on WoSCC data, and the data from other databases need to be converted to WoS data format before they can be analysed, so we finally chose the WoS database as the retrieval source.

**Fig. 1:**
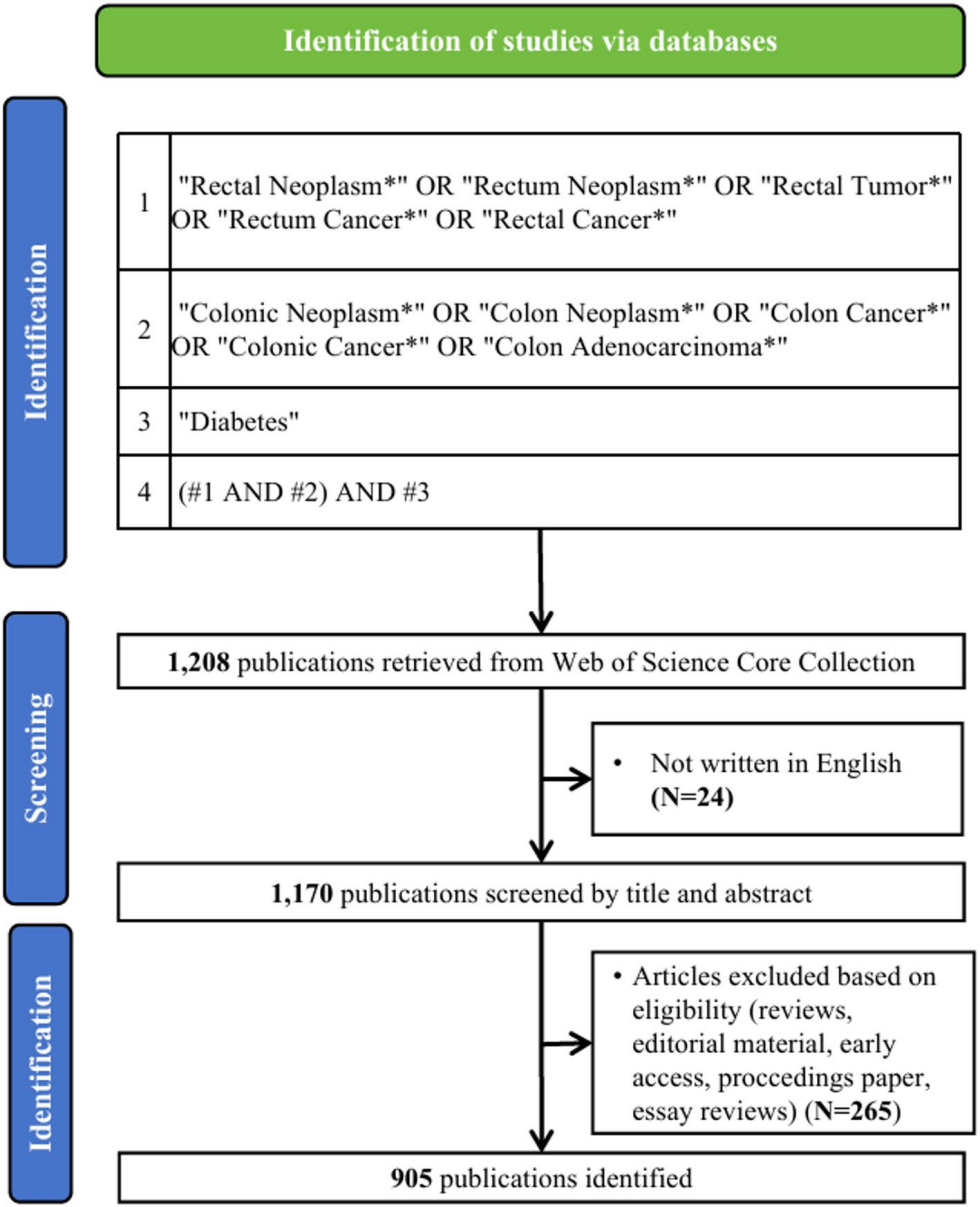
Publications screening flowchart

On July 10, 2024, we performed a comprehensive literature search in WoSCC database. The primary search terms were “Rectal Neoplasm”, “Colonic Neoplasm” and “Diabetes”, with language restricted to English and publication type limited to Articles. The detailed search strategy is provided in Supplementary Table S1. To avoid the impact of database updates, all search results were downloaded within the same day in multiple formats, including plain text, Excel, BibTex, and tab-delimited files. Ultimately, 905 records were identified for analysis. The overall research workflow is schematically illustrated in Fig. 1.

### Data analysis and visualization

The retrieved data were imported into CiteSpace (version 6.3.R3), VOSviewer (version 1.6.20), the bibliometrix package in R (version 4.4.0), Scimago Graphica, and the Bibliometric Analysis Online Platform (https://bibliometric.com/) to perform analysis of published studies and create visual mapping diagrams. VOSviewer enables knowledge mapping visualisation of large-scale literature data through co-authorship analysis, co-occurrence analysis, and co-citation analysis, representing authors, journals, and related entities [17–19]. CiteSpace was employed to conduct country collaboration mapping, journal co-authorship analysis, reference co-citation analysis, and keyword clustering, thereby identifying developmental trajectories and emerging trends within the research domain [19, 20]. We conducted burst detection analysis [21] on the references to identify emerging academic trends and novel disciplinary developments, enabling the projection of frontier research directions and potential hotspots [22]. The keyword timelines [23] delineated temporal ranges of clustered research hotspots, revealing interconnections between distinct thematic concentrations, thereby mapping the evolutionary trajectory of research focuses since the inception of CRC comorbidity studies. Subsequent time-zone maps of keywords demonstrated their chronological distribution patterns, with keyword emergence and evolution elucidating shifts in research themes and trends. An international collaboration network was constructed using the Bibliometric Analysis Online Platform. The historiograph mapping of references was generated using the bibliometrix package in R, which visualizes inter-citation relationships among publications and delineates knowledge dissemination trajectories within the research domain.

## Results

### Analysis of annual publications and trends

**Fig. 2:**
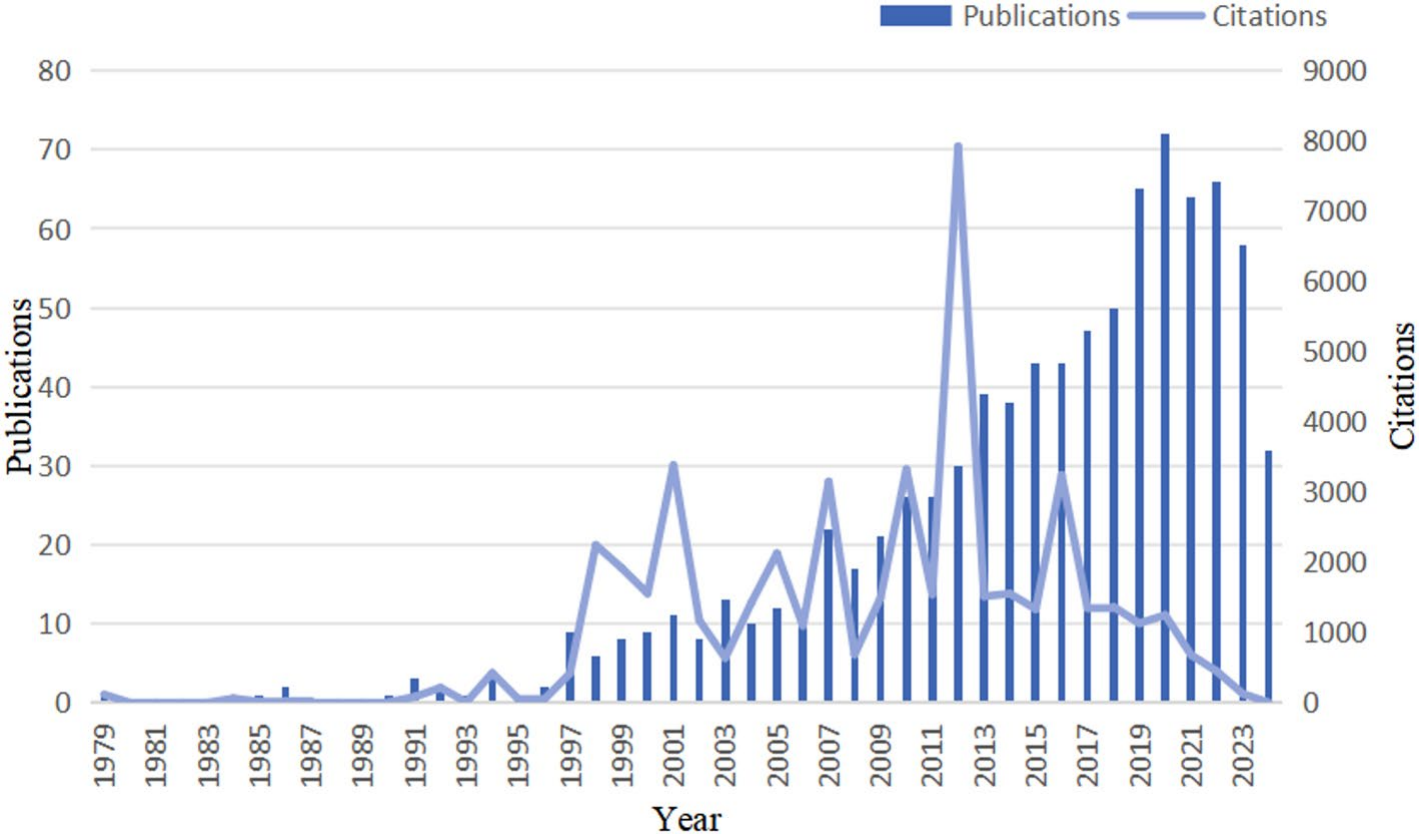
Annual publications and citations

This study conducted a comprehensive analysis of 905 publications meeting predefined inclusion criteria. Figure 2 displays their annual distribution, revealing a steady increase starting from the late 1990s with accelerated growth after 2005, peaking around 2020. Citation counts peaked in 2012, indicating potential high-impact outputs during this period, followed by a moderate decline in citations after 2018.

### Analysis of the trend of countries/regions

**Fig. 3:**
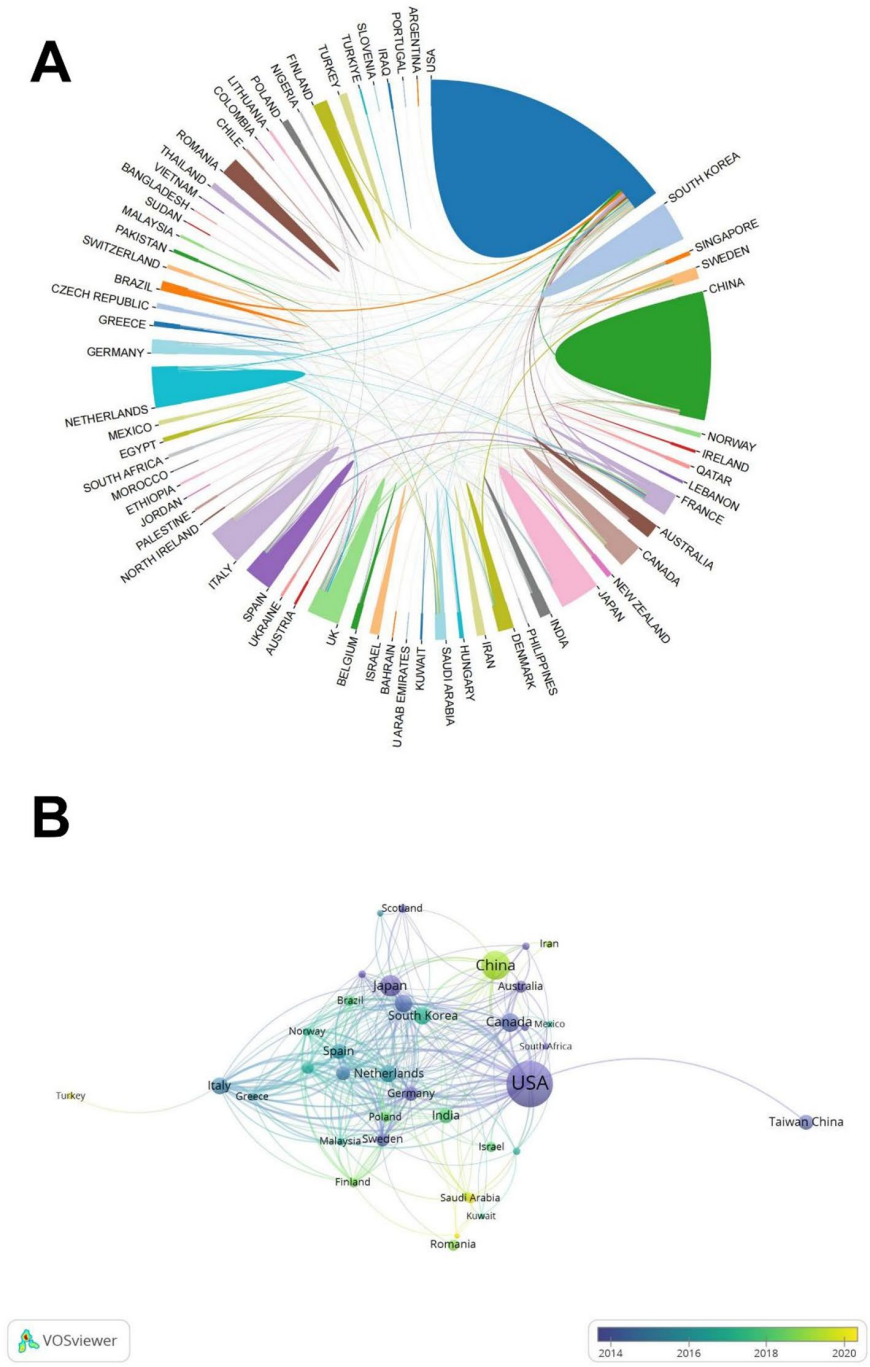
**A** International collaboration network. Automatically removes isolated nodes that do not have cooperative relationships with other countries. **B** Temporal overlay network map of international co-authorship

The 905 publications originated from 72 countries/regions and 1,605 institutions. The United States (308 articles; average citations per article 98.98; centrality 0.32) dominated in publication volume, followed by China (122 articles; average citations per article 15.66; centrality 0.06), but the latter demonstrated relatively lower citation impact and centrality. New Zealand (40 articles; average citations per article 114.75) exhibited the highest citations per article among the top 10 productive countries, succeeded by the United States and the United Kingdom (Table 1). In our research, the cooperative relationship between countries reveals that the United States and other countries/regions cooperate most closely (Fig. 3A and B).

**Table 1:** Top 10 countries/regions in terms of number and centrality of publications related to research on colorectal cancer-diabetes comorbidity

Rank	Publications	Country/region	Average citations	Rank	Centrality	Country/region	Average citations
1	308	United States	98.98	1	0.32	United States	98.98
2	122	China	15.66	2	0.16	United Kingdom	15.66
3	66	Japan	29.79	3	0.13	Canada	57.58
4	52	Canada	57.58	4	0.10	Saudi Arabia	11.29
5	46	England	76.04	5	0.08	France	116.55
6	45	South Korea	26.40	6	0.08	Malaysia	61.22
7	40	Netherlands	114.75	7	0.07	Italy	49.85
8	39	Italy	49.85	8	0.06	Germany	110.86
9	33	Taiwan China	35.76	9	0.06	China	15.66
10	32	Spain	72.56	10	0.06	Spain	72.56

### Analysis of institutions

**Fig. 4:**
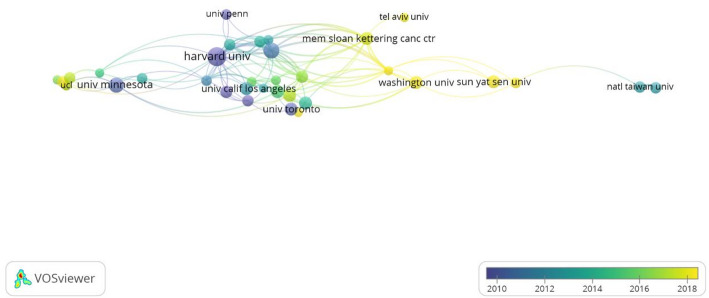
Institution collaboration network diagram

The analysis revealed less frequent inter-institutional collaborations compared to international collaborations, with Harvard University and the Brigham & Women’s Hospital occupying central positions in the network (Fig. 4). This phenomenon may be linked to the prominent scholar Edward Giovannucci, who has affiliations with both institutions. The majority of the top 10 institutions by publication volume were located within the United States (Table 2). Notably, Harvard University ranked first with 28 relevant publications, demonstrating leadership in both total citations and average citations per publication.

**Table 2:** Top 10 institutions by publication volume related to research on colorectal cancer-diabetes comorbidity

Rank	Organization	Country	Publications	Total citations	Average citations
1	Harvard Univ	United States	28	12,052	430.43
2	Brigham & Womens Hosp	United States	19	4643	244.37
3	Univ Minnesota	United States	18	1883	104.61
4	Univ Calif Los Angeles	United States	13	2061	158.54
5	Univ Queensland	Australia	13	2332	179.38
6	Univ Utah	United States	13	343	26.38
7	Mayo Clin	United States	12	496	41.33
8	Mem Sloan Kettering Canc Ctr	United States	12	425	35.42
9	Sun Yat Sen Univ	China	12	259	21.58
10	Univ Toronto	Canada	12	1360	113.33

### Analysis of authors

**Fig. 5:**
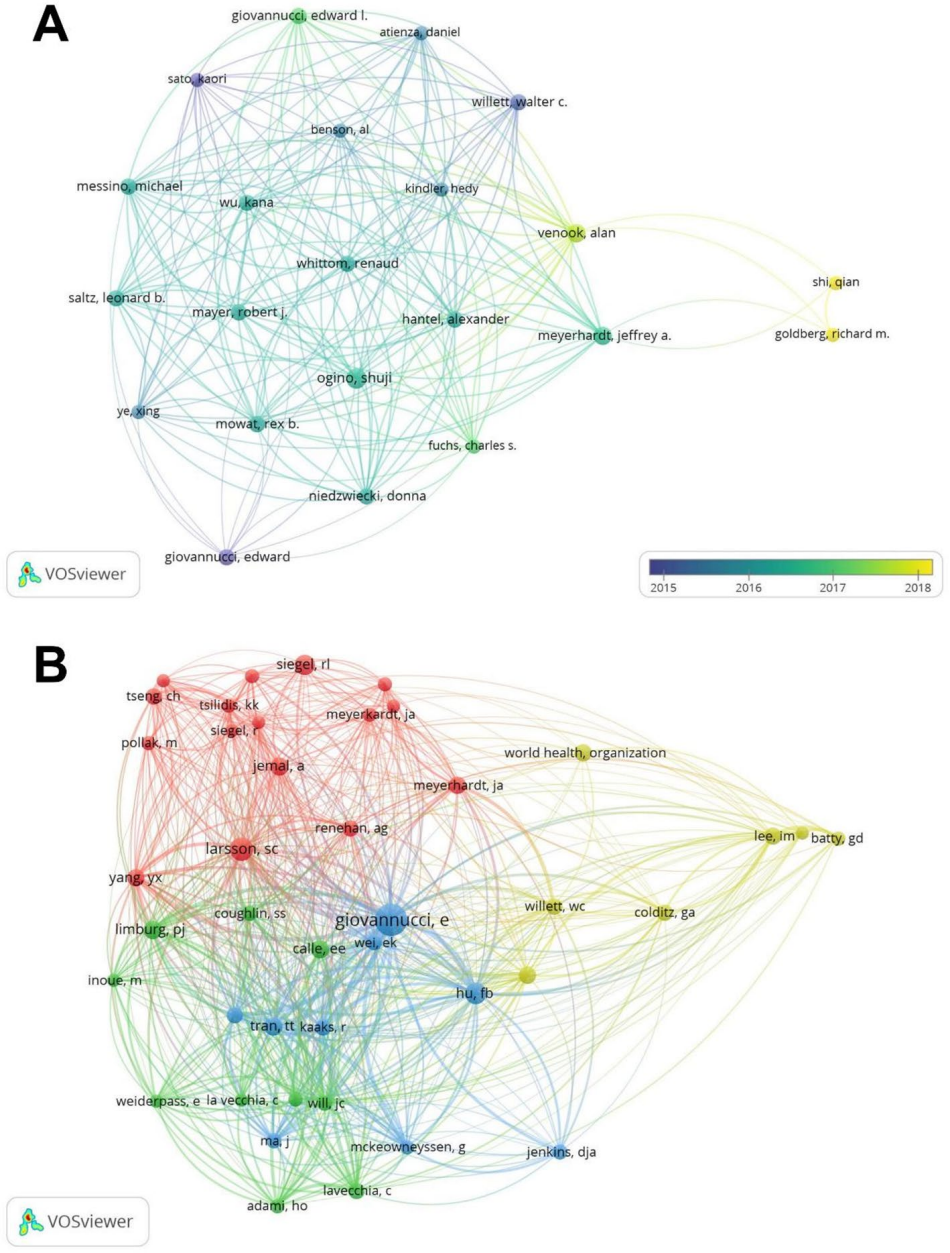
**A** Authors collaboration diagram. **B** Cited author co-occurrence map, different colours in (**B**) distinguish different clusters, and the same colour indicates similarity of studies

In past studies, a total of 5389 researchers have contributed in the field of colorectal cancer-diabetes comorbidity. The top three authors with the most publications were Ogino, Shuji (*n* = 6); Meyerhardt, Jeffrey A. (*n* = 5); and Venook, Alan (*n* = 5). The top three most cited authors were Giovannucci, Edward (*n* = 666); Ogino, Shuji (*n* = 406); and Willett, Walter C. (*n* = 262) (Table 3). We used VOSviewer to generate an author collaboration network map in this research field, revealing that Shi, Qian, and Goldberg, Richard M., have maintained active collaborative partnerships in recent years (Fig. 5A). The co-citation network analysis identified Giovannucci, Edward as occupying the central hub position within the author network (Fig. 5B).

**Table 3:** Top 10 authors in terms of publications and citations related to colorectal cancer-diabetes comorbidity

Rank	Authors (rank by publications)	Publications	Average citations	Rank	Authors (rank by citations)	Citations
1	Ogino, Shuji	6	67.6667	1	Giovannucci, Edward	666
2	Meyerhardt, Jeffrey A.	5	41.4	2	Ogino, Shuji	406
3	Venook, Alan	5	37.4	3	Willett, Walter C.	262
4	Giovannucci, Edward	4	166.5	4	Giovannucci, Edward L.	241
5	Giovannucci, Edward L.	4	60.25	5	Meyerhardt, Jeffrey A.	207
6	Hantel, Alexander	4	46	6	Venook, Alan	187
7	Willett, Walter C.	4	65.5	7	Hantel, Alexander	184
8	Messino, Michael	4	46	8	Mayer, Robert J.	184
9	Mowat, Rex B.	4	46	9	Messino, Michael	184
10	Niedzwiecki, Donna	4	46	10	Mowat, Rex B.	184

### Analysis of journals and subjects

**Fig. 6:**
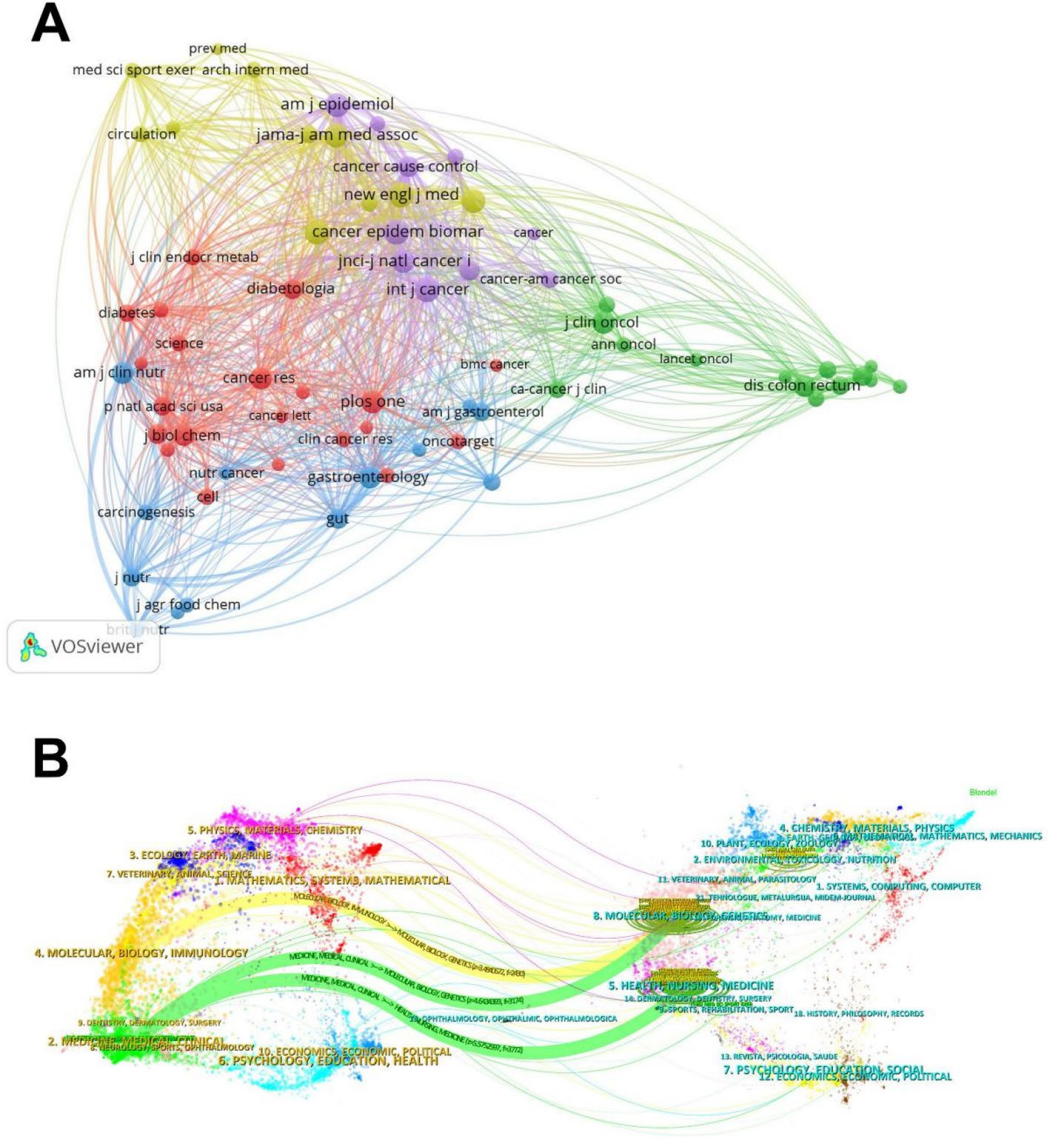
**A** Journal co-citation network. Distinct clusters are differentiated by colour coding, with identical colours indicating similarity in research focus areas. **B** Journal dual-map overlay diagram

A total of 513 journals have published articles related to A total of 513 journals have published articles related to colorectal cancer-diabetes comorbidity. PLOS ONE demonstrated the highest publication output (25 publications, IF = 2.9), followed by the INTERNATIONAL JOURNAL OF COLORECTAL DISEASE ranking second (14 publications, IF = 2.5). Interestingly, the most prolific journals were not necessarily the most cited, with PLOS ONE at the bottom of the citation rankings. THE LANCET received the highest citation count (6,785 citations, IF = 98.4). Notably, nearly half of the top-cited journals were classified in the Q1 JCR quartile, indicating substantial academic value within this research field (Table 4). The journal co-citation network (Fig. 6A) and dual-map overlay diagram (Fig. 6B) collectively visualise the predominant citation pathways between citing journals and cited journals in this field. The three most prominent coloured paths illustrate citation relationships where journals in the Medicine, Medical, Clinical fields reference publications from both the Health, Nursing, Medicine and Molecular, Biology, Genetics fields, followed by citations from Molecular, Biology, Immunology journals to Molecular, Biology, Genetics literature. This pattern highlights interdisciplinary convergence within the field, demonstrating that modern medical research is increasingly integrating knowledge across multiple specialised disciplines.

**Table 4:** Top 10 journals in terms of publications and citations related to colorectal cancer-diabetes comorbidity

Rank	Top 10 most productive journals				Top 10 most–cited journals				
	Journals	Productions	JCR	IF (2023)	Journals	Average citations	Citations	JCR	IF (2023)
1	PloS One	25	Q3	2.9	Lancet	1696.25	6785	Q1	98.4
2	International Journal Of Colorectal Disease	14	Q3	2.5	American Journal Of Clinical Nutrition	313.5	1881	Q1	6.5
3	Cancer Epidemiology Biomarkers & Prevention	11	Q3	3.7	JNCI-Journal Of The National Cancer Institute	298.25	1193	Q1	99.0
4	Diseases Of The Colon & Rectum	10	Q2	3.2	Journal Of Nutrition	248.4	1242	Q3	3.7
5	Cancer Causes & Control	10	Q4	2.2	American Journal Of Epidemiology	205.4286	1438	Q2	5
6	Scientific Reports	10	Q2	3.8	Journal Of Clinical Oncology	145.7143	1020	Q1	42.1
7	Clinical Gastroenterology And Hepatology	9	Q1	11.6	Clinical Gastroenterology And Hepatology	84.1111	757	Q1	11.6
8	American Journal Of Epidemiology	7	Q2	5.0	Cancer Epidemiology Biomarkers & Prevention	72	792	Q3	3.7
9	Journal Of Clinical Oncology	7	Q1	42.1	Diseases Of The Colon & Rectum	70	700	Q2	3.2
10	Cancer	7	Q2	6.1	Plos One	41.24	1031	Q3	2.9

### Analysis of articles and references

**Fig. 7:**
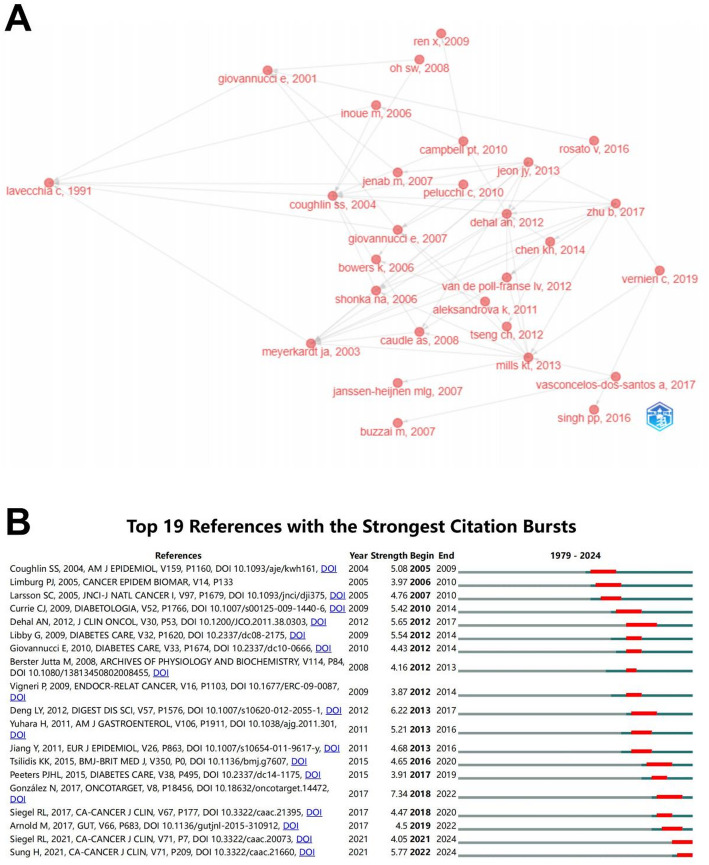
**A** Historical direct citation network. **B** Top 19 references with the strongest citation bursts

To explore changes in the content of relevant studies over time, we analysed the historical direct citation network of colorectal cancer-diabetes comorbidity references (Fig. 7A and Supplementary Table S2). Figure 7B shows the top 19 references with the strongest citation bursts. Among them, Nieves González et al.’s review “2017 update on the relationship between diabetes and CRC: epidemiology, potential molecular mechanisms and therapeutic implications” published in ONCOTARGET (Strength = 7.34) was identified as the reference with the strongest citation burst. And the two most recent high-burst references were both cancer statistical data reports. Table 5 listed the top 10 highly cited references. “Effect of physical inactivity on major non-communicable diseases worldwide: an analysis of burden of disease and life expectancy”, published in Lancet in 2012, was cited 5,268 times [24].

**Table 5:** Top 10 references in terms of citations related to the research on colorectal cancer-diabetes comorbidity

Rank	Title	Citation	Year	Journal	JCR	IF (2023)
1	Effect of physical inactivity on major non-communicable diseases worldwide: an analysis of burden of disease and life expectancy	5268	2012	Lancet	Q1	98.4
2	Lack of exercise is a major cause of chronic diseases	1468	2012	Compr Physiol	Q2	4.2
3	Prebiotic effects: metabolic and health benefits	1430	2010	Br J Nutr	Q3	3.0
4	The economic burden of physical inactivity: a global analysis of major non-communicable diseases	1239	2016	Lancet	Q1	98.4
5	Impact of overweight on the risk of developing common chronic diseases during a 10-year period	1126	2001	Arch Intern Med	-	-
6	Current estimates of the economic cost of obesity in the United States	844	1998	Obes Res	-	-
7	The relation between different dimensions of alcohol consumption and burden of disease: an overview	785	2010	Addiction	Q1	5.2
8	Systemic treatment with the antidiabetic drug metformin selectively impairs p53-deficient tumor cell growth	769	2007	Cancer Res	Q1	12.5
9	Diabetes mellitus and risk of colorectal cancer: a meta-analysis	762	2005	J Natl Cancer Inst	Q1	9.9
10	Physical activity and risk of breast cancer, colon cancer, diabetes, ischemic heart disease, and ischemic stroke events: systematic review and dose-response meta-analysis for the Global Burden of Disease Study 2013	743	2016	BMJ	Q1	93.6

### Analysis of keywords

**Fig. 8:**
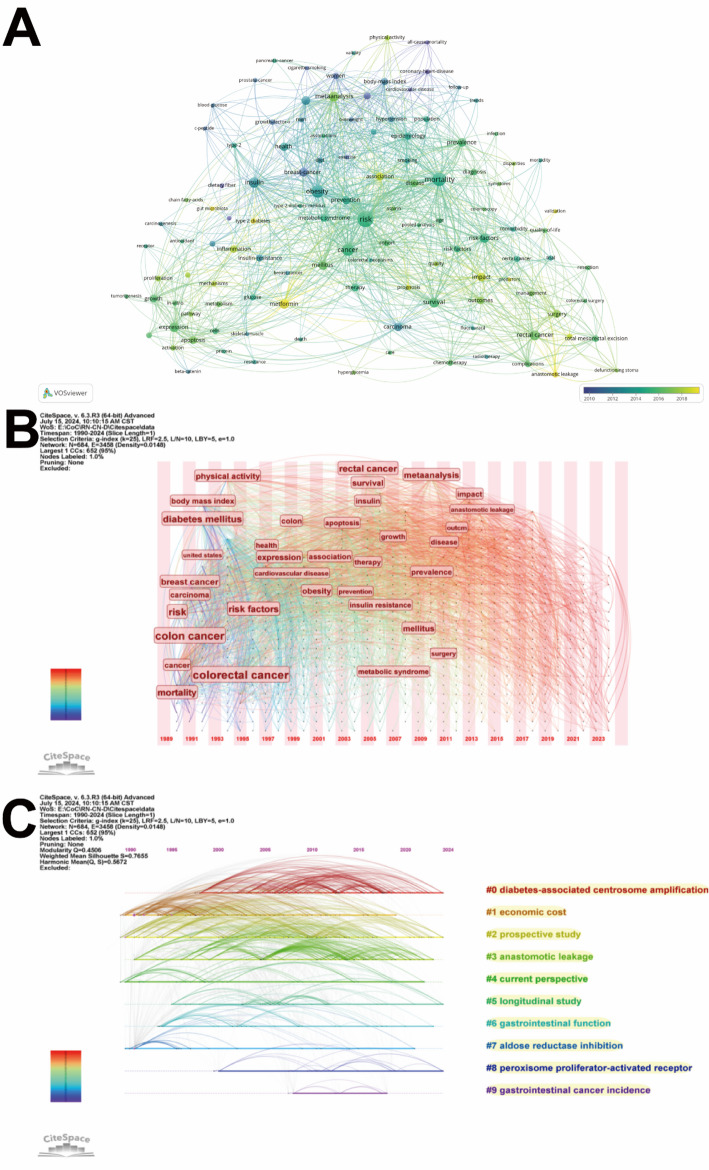
**A** Keyword co-occurrence network with temporal overlay. **B** Keyword time zone map. **C** Keyword timeline map

The keyword co-occurrence network with temporal overlay, generated using VOSviewer, visualises both high-frequency keywords and newly emerged terms in recent years (Fig. 8A). To mitigate visualization bias caused by excessive frequency dominance, the terms “colon cancer,” “rectal cancer,” and “diabetes” were excluded from this analysis due to their disproportionately high occurrence rates in the dataset. The emergence of new keywords since 2018, including “metformin”, “anastomotic leakage”, “gut microbiota”, and “inflammation”, demonstrates a growing research focus on therapeutic prognosis and molecular biological mechanisms in colorectal cancer-diabetes comorbidity. The keyword time zone map produced by Citespace reveals the variation of high-frequency keywords over time (Fig. 8B). The keyword timeline map clearly shows the changes in each research hotspot over the time horizon (Fig. 8C). The timeline map structure included the X-axis representing the publication year and the Y-axis corresponding to the keyword cluster names. The keyword co-occurrence network analysis employing the Log-Likelihood Ratio (LLR) algorithm yielded a modularity (Q) > 0.3, indicating statistically significant clustering, and a silhouette (S) index > 0.7, confirming robust cluster configuration [25]. Our dataset demonstrated clustering metrics of Q = 0.4506 and S = 0.7655, aggregating 10 thematic clusters. We observe that in the recent studies mainly focusing on “#0 diabetes-associated centrosome amplification” (Cluster 0), while “#1 economic cost” (Cluster 1), “#2 prospective study” (Cluster 2), and “#3 anastomotic leakage” (Cluster 3) have remained prominent research directions since the 1990s.

## Discussion

### Research landscape and collaborative networks

To our knowledge, this study represents the first bibliometric analysis in the field of colorectal cancer-diabetes comorbidity, providing essential reference data for researchers intending to explore this field. Since 2005, the number of publications on colorectal cancer-diabetes comorbidity began to trend upward each year, with a peak of about 70 publications in 2020. Citations have likewise been on the rise overall. Beginning in the late 1990s, there were several more significant spikes in citations. In 2010, a consensus report from a joint conference in the field of oncology and diabetes stated that T2DM is a risk factor for breast cancer, CRC, and many other site-specific tumours [26]. The global trend in the quantity and impact of publications and citations on colorectal cancer and diabetes has followed a marked upward trajectory, reflecting growing academic interest and the increasing relevance of this research area.

At the national level, both the United States and China show stronger preferences for international collaboration in colorectal cancer-diabetes comorbidity research. China demonstrates remarkable publication volume and recent research activity in this field, making substantial contributions. Over the past 20 years, China has gradually established chronic disease management frameworks and enhanced its universal health insurance system [27, 28]. Specialized diabetes and cancer surveillance and registration databases have been implemented across all regions. The improvement of these medical systems reveals that China has the basis for evaluating the geographical differences of colorectal cancer diabetes complications, and can carry out multi center research on the mechanism of disease progression in the future.

The top 10 institutions by publication volume - about 80% belong to North America. Harvard University is the most widely published institution in this field, maintaining close collaborations with the Brigham & Women’s Hospital. The cooperation among these global high-level research institutions can effectively promote knowledge exchange and accelerate the output of innovative achievements in this field by sharing their research findings. Therefore, strengthening the cooperation between institutions is very important for the future development of this field. Professor Edward Giovannucci, holding joint appointments at both institutions, has the highest total citations of 666, with an average citation of 166.5, and has pioneered the field of cancer prevention by pointing out that dietary and lifestyle factors greatly influence tumorigenesis. Diet-influenced insulin, insulin-like growth factor-1 (IGF-1) and inflammatory factors can induce tumour development. Insulin is also a mitogen for tumour cell growth in vitro, which induces activation of the proto-oncogene *ras* and sustained activation of the MAPK pathway, ultimately contributing to colorectal carcinogenesis [29]. This reveals that exploring dietary differences via diabetes to initiate CRC is a valuable direction for future research.

This field demonstrates robust interdisciplinary integration, primarily spanning medicine, molecular biology, and clinical practice while intersecting with computer science, mathematics, physics, socioeconomics, and education sciences. The current research environment encourages participation from non-biomedical disciplines to promote interdisciplinary collaboration to continuously improve the diverse academic framework.

### Emerging thematic clusters and their research implications

Keyword clustering analysis identified three prominent and evolving research frontiers, each presenting distinct mechanistic questions and clinical translation opportunities. Diabetes-associated centrosome amplification (CA), anastomotic leakage (AL), and metformin have gained significant attention in recent years and have revealed key areas for future research. Diabetes-associated centrosome amplification has emerged as the most intensively investigated focus in the colorectal cancer–diabetes comorbidity field since 2000, indicating novel mechanistic breakthroughs in pathogenesis elucidation. The sustained academic attention on anastomotic leakage highlights researchers’ extensive clinical trial investigations into postoperative complications among patients with colorectal cancer–diabetes comorbidity. Since 2018, research interest in metformin has increased significantly, reflecting its dual therapeutic potential in glycaemic regulation and modulation of neoplastic pathways.

#### Diabetes-associated centrosome amplification: a mechanistic frontier

Cluster #0 underscores “diabetes-associated centrosome amplification (CA)” as a novel and intense research focus. Theodor Boveri first postulated the potential tumorigenic effects of CA [30]. Genetic modification in cellular and animal models has provided compelling evidence demonstrating a causal relationship between CA and both tumorigenesis and metastatic progression [31–34]. Taking T2DM as an example, it was found not only to increase the risk of CRC incidence and metastasis rates [26], but also to induce CA in cancer-susceptible epithelial cells (e.g., intestinal polyps) and established colorectal carcinoma cells [35]. In vitro experimentation demonstrates that chronic exposure of non-neoplastic colonic epithelium to advanced glycation end products (AGEs) initiates tumorigenesis in immunocompromised murine models—an oncogenic process effectively abrogated through pharmacological inhibition or genetic silencing of CA-associated signalling pathways [35, 36]. However, critical knowledge gaps persist: the upstream triggers within the diabetic environment (e.g., specific AGEs, oxidative stress species) are not fully defined; the causal role of CA in diabetes-promoted CRC progression in vivo requires further validation; and the translational potential of CA as a biomarker for early detection or as a therapeutic target remains unexplored. Future research should: (i) employ multi-omics approaches to delineate the complete signaling cascade from hyperglycemia to CA; (ii) investigate the clinical utility of CA assessment in polyps or liquid biopsies from diabetic patients for risk stratification.

#### Anastomotic leakage in colorectal cancer-diabetes patients: a clinical challenge

While chemotherapeutic agents, immunotherapies, and molecular-targeted drugs have demonstrated unprecedented progress in CRC, radical surgical resection maintains its gold-standard status for early-stage CRC cases meeting R0 criteria (tumour-free margin ≥ 1 mm histologically confirmed). Standardised surgical protocols for colorectal malignancies, including radical colectomy (RC), low anterior resection (LAR) for rectal cancer, and intersphincteric resection (ISR), necessarily require the implementation of precision anastomotic techniques - encompassing end-to-end, end-to-side, and side-to-side configurations. AL is one of the serious complications that can occur after gastrointestinal surgery, which can have a significant negative impact on the survival and quality of life of postoperative patients [37, 38]. Prolonged hyperglycemia will result in delayed wound healing, and AL is about three times more likely to occur in patients with T2DM than in those without T2DM [39, 40]. Fibroblasts and endothelial cells constitute the primary cellular components of granulation tissue [41], with fibroblasts mediating wound contraction through matrix deposition [41, 42], while endothelial cells facilitate angiogenesis during the proliferative phase of healing [43, 44]. Hyperglycemia amplifies intracellular oxidative stress by activating the classical AGE-RAGE axis [45] and through atypical interactions between 3-deoxyglucosone (3-DG) and integrins alpha-1/beta-1 (α1β1) [46]. Reactive oxygen species (ROS) generated in mitochondria stimulate multiple downstream signalling pathways, including p38/MAPK, Forkhead box protein O1 (FOXO1), and nuclear factor-kappa B (NF-κB) [47–49]. This cascade triggers inflammatory responses and promotes cellular senescence and apoptosis. The ensuing inflammatory response is accompanied by M1 macrophage polarization, which impairs fibroblasts and inhibits their ability to secrete extracellular matrix (ECM) [50]. Concurrently, the presence of ROS and proinflammatory cytokines coincides with increased expression of matrix metalloproteinases (MMPs)—a family of zinc-dependent proteolytic enzymes capable of degrading protein components within the ECM [51, 52]. When ECM levels persistently fall below the threshold required for wound healing, fibroblast migration, anchorage, and proliferation become significantly impaired [53]. In addition, hyperglycemia impairs the angiogenic function of endothelial cells through ROS-mediated endothelial nitric oxide synthase (e-NOS) inhibition [54–56], activation of the AGE-RAGE axis [57], and release of pro-inflammatory factors [58]. Diabetic wounds are characterized by a chronic low-grade inflammatory state, insufficient ECM deposition, aberrant angiogenesis, and impaired contractile function (reduced α-smooth muscle actin (α-SMA) expression) [59]. Elevated levels of tumour necrosis factor-α (TNF-α) and reduced transforming growth factor-β1 (TGF-β1) contribute to an imbalance in repair signalling, promoting apoptosis and increased protease activity [59]. These complex pathophysiological processes affect the formation of granulation tissue, which is the key factor of AL after colorectal cancer surgery. Despite this understanding, translational gaps hinder clinical improvement: perioperative glycemic management protocols are not standardized for CRC surgery; reliable predictive biomarkers for AL risk in diabetic patients are lacking; and targeted interventions to improve wound healing in this cohort are underdeveloped. Future studies must prioritize: (i) prospective trials to optimize perioperative glucose control strategies; (ii) discovery and validation of immune-metabolic biomarkers—such as specific MMPs and cytokine profiles—predictive of AL; (iii) development of novel adjunctive therapies, including targeted antioxidants and growth factor applications, to enhance anastomotic healing in patients with diabetes.

#### Metformin in colorectal cancer-diabetes comorbidity: therapeutic promise and uncertainty

Metformin, the first-line oral antihyperglycemic agent for T2DM, exerts its therapeutic effects through mitochondrial respiratory chain complex I/IV inhibition. This bioenergetic interference elevates cellular AMP/ATP ratios, subsequently activating lysosomal AMP-activated protein kinase (AMPK) signalling cascades that coordinate hepatic gluconeogenesis suppression and peripheral glucose uptake enhancement [60]. Moreover, its potential cancer-preventive and anti-neoplastic effects have garnered increasing attention in the field of CRC [61]. Current evidence reveals two interconnected mechanistic aspects: (1) direct regulation through signalling pathways including mTOR, PI3K/AKT/mTOR (PAM), and K-Ras, and (2) indirect metabolic modulation involving insulin sensitivity and glucose metabolism [62, 63]. These dual mechanisms exhibit functional crosstalk rather than operating independently. Buzzai et al. demonstrated that metformin induces energy stress through the AMPK-p53 pathway, forcing tumour cells to undergo metabolic conversion (such as enhanced fatty acid β-oxidation and autophagy), while p53-deficient colon cancer cells undergo apoptosis due to their inability to adapt to this stress [64]. However, some studies have reveals less than satisfactory outcomes regarding its protective effects in diabetic patients with CRC [65]. The 2016 N0147 trial conducted by the North Central Cancer Treatment Group in the United States revealed no significant differences in disease-free survival (DFS), overall survival (OS), or time to recurrence (TTR) between metformin users and non-users among stage III colon cancer patients undergoing radical resection. This lack of benefit persisted regardless of KRAS or BRAF mutation status or mismatch repair (MMR) proficiency [66]. This discrepancy underscores a critical research gap: the lack of predictive biomarkers to identify patient subsets who might benefit from metformin, such as those defined by p53 status, KRAS/BRAF mutations, or metabolic profile. Furthermore, metformin’s interaction with chemotherapy and immunotherapy regimens remains poorly understood. To address these issues, future research directions should include the following: (i) conducting prospective clinical trials stratified by biomarkers (e.g., p53 and MMR status) to resolve efficacy controversies; (ii) performing preclinical and clinical studies evaluating metformin in combination with immunotherapy treatments, given its immunomodulatory potential; (iii) carrying out pharmacokinetic and pharmacodynamic studies to determine whether anticancer effects require doses different from those used for glycemic control.

### Shifts in research focus: from epidemiology to mechanisms and translation

The temporal analysis of keywords and references reveals a clear paradigm shift. Early research (pre-2000s) was dominated by establishing epidemiological links. Subsequent decades saw a rise in investigating intermediate mechanisms like hyperinsulinemia and Metabolic Syndrome (MetS). Currently, the frontier is characterized by a focus on molecular mechanisms (e.g., CA, gut microbiota) and therapeutic/clinical outcomes (e.g., AL, metformin, survival). This evolution aligns with the broader movement towards precision oncology. However, a significant translational gap remains: few studies integrate patient-derived multi-omics data to personalize risk prediction or treatment for the colorectal cancer-diabetes comorbidity population. Bridging this gap requires interdisciplinary consortia that combine deep phenotyping, molecular profiling, and clinical trial data.

### Limitation

This study performed a bibliometric analysis of literature on colorectal cancer-diabetes comorbidity. Unlike traditional reviews, this approach offers a clearer visualization of research progress. However, several limitations exist. First, relying solely on the WoSCC database may introduce bias due to incomplete coverage, particularly regarding non-English or regional publications, though WoSCC remains a widely authoritative source. Second, using citation metrics to assess impact is inherently biased: highly cited articles are not always the most rigorous, and significant newer work may lack sufficient citations. Third, keyword clustering and thematic mapping depend on algorithmic parameters and thresholds; varying these settings could alter results, underscoring the sensitivity of such unsupervised methods. Finally, bibliometric analysis is descriptive and correlational—it cannot establish causality or evaluate the scientific validity of findings. While it identifies trends, collaborations, and emerging topics, it does not assess evidence quality. Thus, these conclusions should be complemented by systematic reviews and experimental studies.

## Conclusion

In this study, we conducted bibliometric and visual analysis with CiteSpace visualisation to map the colorectal cancer-diabetes comorbidity research landscape, which is more intuitively and clearly reflected in the research progress in the target area when compared with traditional narrative reviews. Colorectal cancer-diabetes comorbidity, as an interdisciplinary field of great interest, has research potential in the future.

## Supplementary Information

Below is the link to the electronic supplementary material.

Supplementary Material 1

## Data Availability

The bibliometric data used in this study were retrieved from the publicly accessible database “Web of Science”, with the search completed on July 10, 2024. The search strategy and inclusion/exclusion criteria are detailed in the MATERIALS AND METHODS section. Due to database license agreements and restrictions on redistribution, the raw bibliometric records cannot be publicly shared. However, all processed data and analysis scripts (e.g., for data cleaning, citation network construction) are available from the corresponding author upon reasonable request via email at [13848018266@163.com](mailto:13848018266@163.com).

## Abbreviations

CRC: Colorectal cancer
DM: Diabetes mellitus
WoSCC: The Web of Science Core Collection
T2DM: Type 2 diabetes mellitus
IDF: The International Diabetes Federation
AJCC: The American Joint Committee on Cancer
LLR: The Log-Likelihood Ratio
IGF-1: Insulin-like growth factor-1
MetS: Metabolic Syndrome
AL: Anastomotic leakage
CA: Centrosome amplification
AGEs: Advanced glycation end products
RC: Radical colectomy
LAR: Low anterior resection
ISR: Intersphincteric resection
ROS: Reactive oxygen species
FOXO1: Forkhead box protein O1
NF-κB: Nuclear factor-kappa B
ECM: Extracellular matrix
MMPs: Matrix metalloproteinases
e-NOS: Endothelial nitric oxide synthase
α-SMA: α-smooth muscle actin
TNF-α: Tumour necrosis factor-α
TGF-β1: Transforming growth factor-β1
AMPK: AMP-activated protein kinase
PAM: PI3K/AKT/mTOR
DFS: Disease-free survival
OS: Overall survival
TTR: Time to recurrence
MMR: Mismatch repair

## References

[1] King H, Aubert RE, Herman WH. Global burden of diabetes, 1995–2025: prevalence, numerical estimates, and projections. Diabetes Care. 1998;21(9):1414–31. 9727886 10.2337/diacare.21.9.1414

[2] Klimeck L, Heisser T, Hoffmeister M, et al. Colorectal cancer: A health and economic problem. Best Pract Res Clin Gastroenterol. 2023;66:101839. 37852707 10.1016/j.bpg.2023.101839

[3] Ternák G, Németh M, Rozanovic M, et al. Antibiotic consumption patterns in European countries might be associated with the prevalence of type 1 and 2 diabetes. Front Endocrinol (Lausanne). 2022;13:870465. 35600582 10.3389/fendo.2022.870465 PMC9120822

[4] Saeedi P, Petersohn I, Salpea P, et al. Global and regional diabetes prevalence estimates for 2019 and projections for 2030 and 2045: results from the international diabetes federation diabetes Atlas, 9(th) edition. Diabetes Res Clin Pract. 2019;157:107843. 31518657 10.1016/j.diabres.2019.107843

[5] Dekker E, Tanis PJ, Vleugels JLA, et al. Colorectal Cancer Lancet. 2019;394(10207):1467–80. 31631858 10.1016/S0140-6736(19)32319-0

[6] Medema JP. Cancer stem cells: the challenges ahead. Nat Cell Biol. 2013;15(4):338–44. 23548926 10.1038/ncb2717

[7] Bray F, Laversanne M, Sung H, et al. Global cancer statistics 2022: GLOBOCAN estimates of incidence and mortality worldwide for 36 cancers in 185 countries. CA Cancer J Clin. 2024;74(3):229–63. 38572751 10.3322/caac.21834

[8] Amin Mb ES, Greene F, editors. Et al., Editors.American joint committee on cancer (AJCC) cancer staging manual. 8th ed. New York: Springer International; 2017.

[9] Berrino F, Gatta G, Chessa E, et al. Introduction: the EUROCARE II study. Eur J Cancer. 1998;34:2139–53. (14 Spec No). 10070280 10.1016/s0959-8049(98)00334-7

[10] Larsson SC, Orsini N, Wolk A. Diabetes mellitus and risk of colorectal cancer: a meta-analysis. J Natl Cancer Inst. 2005;97(22):1679–87. 16288121 10.1093/jnci/dji375

[11] Deng L, Gui Z, Zhao L, et al. Diabetes mellitus and the incidence of colorectal cancer: an updated systematic review and meta-analysis. Dig Dis Sci. 2012;57(6):1576–85. 22350783 10.1007/s10620-012-2055-1

[12] Singh S, Earle CC, Bae SJ, et al. Incidence of diabetes in colorectal cancer survivors. J Natl Cancer Inst. 2016;108(6):djv402. 26839345 10.1093/jnci/djv402

[13] Kim YI. .Diet, lifestyle, and colorectal cancer: is hyperinsulinemia the missing link? Nutr Rev. 1998;56(9):275–9. 9763878 10.1111/j.1753-4887.1998.tb01765.x

[14] Aleksandrova K, Nimptsch K, Pischon T. Influence of obesity and related metabolic alterations on colorectal cancer risk. Curr Nutr Rep. 2013;2(1):1–9. 23396857 10.1007/s13668-012-0036-9 PMC3562548

[15] Rao Kondapally Seshasai S, Kaptoge S, Thompson A, et al. Diabetes mellitus, fasting glucose, and risk of cause-specific death. N Engl J Med. 2011;364(9):829–41. 21366474 10.1056/NEJMoa1008862 PMC4109980

[16] Thompson DF. Walker C K.A descriptive and historical review of bibliometrics with applications to medical sciences. Pharmacotherapy. 2015;35(6):551–9. 25940769 10.1002/phar.1586

[17] Qu F, Wang G, Wen P, et al. Knowledge mapping of immunotherapy for breast cancer: A bibliometric analysis from 2013 to 2022. Hum Vaccin Immunother. 2024;20(1):2335728. 38563136 10.1080/21645515.2024.2335728 PMC10989689

[18] Van Eck NJ, Waltman L. Software survey: VOSviewer, a computer program for bibliometric mapping. Scientometrics. 2010;84(2):523–38. 20585380 10.1007/s11192-009-0146-3 PMC2883932

[19] Moral-Muñoz JA, Herrera-Viedma E, Santisteban-Espejo A, et al. Software tools for conducting bibliometric analysis in science: an up-to-date review. El Profesional de la Información. 2020;29:1.

[20] Chen C. Searching for intellectual turning points: progressive knowledge domain visualization. Proc Natl Acad Sci U S A. 2004;101(Suppl 1):5303–10. 14724295 10.1073/pnas.0307513100 PMC387312

[21] Kim MC, Nam S, Wang F, et al. Mapping scientific landscapes in UMLS research: a scientometric review. J Am Med Inf Assoc. 2020;27(10):1612–24. 10.1093/jamia/ocaa107 PMC7647344 33059367

[22] Chen C, Dubin R, Kim MC. .Emerging trends and new developments in regenerative medicine: a scientometric update (2000–2014). Expert Opin Biol Ther. 2014;14(9):1295–317. 25077605 10.1517/14712598.2014.920813

[23] Yuan J, Liu Y, Zhang T, et al. Traditional Chinese medicine for breast cancer treatment: a bibliometric and visualization analysis. Pharm Biol. 2024;62(1):499–512. 38813803 10.1080/13880209.2024.2359105 PMC11141317

[24] Lee IM, Shiroma EJ, Lobelo F, et al. Effect of physical inactivity on major non-communicable diseases worldwide: an analysis of burden of disease and life expectancy. Lancet. 2012;380(9838):219–29. 22818936 10.1016/S0140-6736(12)61031-9 PMC3645500

[25] Chen C, Leydesdorff L. Patterns of connections and movements in dual-map overlays: a new method of publication portfolio analysis[J]. Journal of the Association for Information Science and Technology. 2014;65(2):334-351.

[26] Giovannucci E, Harlan D M, Archer M C, et al. Diabetes and Cancer: a Consensus Report[J]. CA: A Cancer Journal for Clinicians. 2010;60(4):207-221. 10.3322/caac.20078 20554718

[27] Ren X, Zhang X, Zhang X, et al. Type 2 diabetes mellitus associated with increased risk for colorectal cancer: evidence from an international ecological study and population-based risk analysis in China. Public Health. 2009;123(8):540–4. 19664792 10.1016/j.puhe.2009.06.019

[28] Jiang R, Xin Y, Peng S, et al. Facilitators and barriers to chronic non-communicable disease management under family Doctor contracting services in China. Front Med (Lausanne). 2025;12:1506016. 40134919 10.3389/fmed.2025.1506016 PMC11932984

[29] Giovannucci E. Insulin and colon cancer. Cancer Causes Control. 1995;6(2):164–79. 7749056 10.1007/BF00052777

[30] Boveri T. Ueber mehrpolige Mitosen Als mittel Zur analyse des Zellkerns. Verh Phys Med Ges Würzburg. 1902;35:67–90.

[31] Basto R, Brunk K, Vinadogrova T, et al. Centrosome amplification can initiate tumorigenesis in flies. Cell. 2008;133(6):1032–42. 18555779 10.1016/j.cell.2008.05.039 PMC2653712

[32] Li J, Xuan JW, Khatamianfar V, et al. SKA1 over-expression promotes centriole over-duplication, centrosome amplification and prostate tumourigenesis. J Pathol. 2014;234(2):178–89. 24827423 10.1002/path.4374

[33] Levine MS, Bakker B, Boeckx B, et al. Centrosome amplification is sufficient to promote spontaneous tumorigenesis in mammals. Dev Cell. 2017;40(3):313–e3225. 28132847 10.1016/j.devcel.2016.12.022 PMC5296221

[34] Dionne LK, Shim K, Hoshi M, et al. Centrosome amplification disrupts renal development and causes cystogenesis. J Cell Biol. 2018;217(7):2485–501. 29895697 10.1083/jcb.201710019 PMC6028550

[35] Li YF, Shi LJ, Wang P et al. Binding between ROCK1 and DCTN2 triggers diabetes‑associated centrosome amplification in colon cancer cells. Oncol Rep. 2021;46(1):151. 10.3892/or.2021.8102 PMC8185503 34080666

[36] He QJ, Wang P, Liu QQ, et al. Secreted Wnt6 mediates diabetes-associated centrosome amplification via its receptor FZD4. Am J Physiol Cell Physiol. 2020;318(1):C48–62. 31618077 10.1152/ajpcell.00091.2019

[37] Lawler J, Choynowski M, Bailey K, et al. Meta-analysis of the impact of postoperative infective complications on oncological outcomes in colorectal cancer surgery. BJS Open. 2020;4(5):737–47. 32525280 10.1002/bjs5.50302 PMC7528523

[38] Koedam TWA, Bootsma BT, Deijen CL, et al. Oncological outcomes after anastomotic leakage after surgery for colon or rectal cancer: increased risk of local recurrence. Ann Surg. 2022;275(2):e420–7. 32224742 10.1097/SLA.0000000000003889

[39] Golightly LK, Jones MA, Hamamura DH, et al. Management of diabetes mellitus in hospitalized patients: efficiency and effectiveness of sliding-scale insulin therapy. Pharmacotherapy. 2006;26(10):1421–32. 16999652 10.1592/phco.26.10.1421

[40] Yao W, Meng Y, Lu M, et al. Impact of type 2 diabetes mellitus on short-term and long-term outcomes of patients with esophageal squamous cell cancer undergoing resection: a propensity score analysis. Cancer Commun (Lond). 2018;38(1):14. 29764483 10.1186/s40880-018-0275-2 PMC5993151

[41] Tefft JB, Chen CS, Eyckmans J. Reconstituting the dynamics of endothelial cells and fibroblasts in wound closure. APL Bioeng. 2021;5(1):016102. 33511324 10.1063/5.0028651 PMC7817247

[42] Tanaka E, Ase K, Okuda T, et al. Mechanism of acceleration of wound healing by basic fibroblast growth factor in genetically diabetic mice. Biol Pharm Bull. 1996;19(9):1141–8. 8889031 10.1248/bpb.19.1141

[43] Toyoda M, Takayama H, Horiguchi N, et al. Overexpression of hepatocyte growth factor/scatter factor promotes vascularization and granulation tissue formation in vivo. FEBS Lett. 2001;509(1):95–100. 11734213 10.1016/s0014-5793(01)03126-x

[44] Singh AK, Sharma A, Warren J, et al. Picroliv accelerates epithelialization and angiogenesis in rat wounds. Planta Med. 2007;73(3):251–6. 17318779 10.1055/s-2007-967119

[45] Giacco F, Brownlee M. Oxidative stress and diabetic complications. Circ Res. 2010;107(9):1058–70. 21030723 10.1161/CIRCRESAHA.110.223545 PMC2996922

[46] Loughlin DT, Artlett CM. Precursor of advanced glycation end products mediates ER-stress-induced caspase-3 activation of human dermal fibroblasts through NAD(P)H oxidase 4. PLoS ONE. 2010;5(6):e11093. 20559423 10.1371/journal.pone.0011093 PMC2885413

[47] Siqueira MF, Li J, Chehab L, et al. Impaired wound healing in mouse models of diabetes is mediated by TNF-alpha dysregulation and associated with enhanced activation of forkhead box O1 (FOXO1). Diabetologia. 2010;53(2):378–88. 19902175 10.1007/s00125-009-1529-y PMC3130195

[48] Sivitz WI, Yorek MA. .Mitochondrial dysfunction in diabetes: from molecular mechanisms to functional significance and therapeutic opportunities. Antioxid Redox Signal. 2010;12(4):537–77. 19650713 10.1089/ars.2009.2531 PMC2824521

[49] Gloire G, Legrand-Poels S, Piette J. NF-kappaB activation by reactive oxygen species: fifteen years later. Biochem Pharmacol. 2006;72(11):1493–505. 16723122 10.1016/j.bcp.2006.04.011

[50] Louiselle AE, Niemiec SM, Zgheib C, et al. Macrophage polarization and diabetic wound healing. Transl Res. 2021;236:109–16. 34089902 10.1016/j.trsl.2021.05.006

[51] Chang M. Restructuring of the extracellular matrix in diabetic wounds and healing: A perspective. Pharmacol Res. 2016;107:243–8. 27033051 10.1016/j.phrs.2016.03.008

[52] Nagase H, Visse R, Murphy G. Structure and function of matrix metalloproteinases and timps. Cardiovasc Res. 2006;69(3):562–73. 16405877 10.1016/j.cardiores.2005.12.002

[53] Xue SN, Lei J, Yang C et al. The biological behaviors of rat dermal fibroblasts can be inhibited by high levels of MMP9. Exp Diabetes Res. 2012;2012:494579. 10.1155/2012/494579 PMC3346983 22577368

[54] Cooke CL, Davidge ST. .Peroxynitrite increases iNOS through NF-kappaB and decreases Prostacyclin synthase in endothelial cells. Am J Physiol Cell Physiol. 2002;282(2):C395–402. 11788351 10.1152/ajpcell.00295.2001

[55] Reinhard H, Jacobsen PK, Lajer M, et al. Multifactorial treatment increases endothelial progenitor cells in patients with type 2 diabetes. Diabetologia. 2010;53(10):2129–33. 20607514 10.1007/s00125-010-1843-4

[56] Lin F, Yang Y, Wei S, et al. Hydrogen sulfide protects against high Glucose-Induced human umbilical vein endothelial cell injury through activating PI3K/Akt/eNOS pathway. Drug Des Devel Ther. 2020;14:621–33. 32103904 10.2147/DDDT.S242521 PMC7027865

[57] Simons M. Angiogenesis, arteriogenesis, and diabetes: paradigm reassessed? J Am Coll Cardiol. 2005;46(5):835–7. 16139133 10.1016/j.jacc.2005.06.008

[58] Domingueti CP, Dusse LM, Carvalho M, et al. Diabetes mellitus: the linkage between oxidative stress, inflammation, hypercoagulability and vascular complications. J Diabetes Complications. 2016;30(4):738–45. 26781070 10.1016/j.jdiacomp.2015.12.018

[59] Goldberg MT, Han YP, Yan C, et al. TNF-alpha suppresses alpha-smooth muscle actin expression in human dermal fibroblasts: an implication for abnormal wound healing. J Invest Dermatol. 2007;127(11):2645–55. 17554369 10.1038/sj.jid.5700890 PMC2366884

[60] Rena G, Hardie DG, Pearson ER. .The mechanisms of action of Metformin. Diabetologia. 2017;60(9):1577–85. 28776086 10.1007/s00125-017-4342-z PMC5552828

[61] Umezawa S, Higurashi T, Komiya Y, et al. Chemoprevention of colorectal cancer: Past, present, and future. Cancer Sci. 2019;110(10):3018–26. 31361372 10.1111/cas.14149 PMC6778640

[62] Rosato V, Tavani A, Gracia-Lavedan E, et al. Type 2 Diabetes, antidiabetic Medications, and colorectal cancer risk: two Case-Control studies from Italy and Spain. Front Oncol. 2016;6:210. 27766252 10.3389/fonc.2016.00210 PMC5052265

[63] Mills KT, Bellows CF, Hoffman AE, et al. Diabetes mellitus and colorectal cancer prognosis: a meta-analysis. Dis Colon Rectum. 2013;56(11):1304–19. 24105007 10.1097/DCR.0b013e3182a479f9 PMC3800045

[64] Buzzai M, Jones RG, Amaravadi RK, et al. Systemic treatment with the antidiabetic drug Metformin selectively impairs p53-deficient tumor cell growth. Cancer Res. 2007;67(14):6745–52. 17638885 10.1158/0008-5472.CAN-06-4447

[65] Vernieri C, Galli F, Ferrari L, et al. Impact of Metformin use and diabetic status during adjuvant Fluoropyrimidine-Oxaliplatin chemotherapy on the outcome of patients with resected colon cancer: A TOSCA study subanalysis. Oncologist. 2019;24(3):385–93. 30606884 10.1634/theoncologist.2018-0442 PMC6519752

[66] Singh PP, Shi Q, Foster NR, et al. Relationship between Metformin use and recurrence and survival in patients with resected stage III colon cancer receiving adjuvant chemotherapy: results from North central cancer treatment group N0147 (Alliance). Oncologist. 2016;21(12):1509–21. 27881709 10.1634/theoncologist.2016-0153 PMC5153338

