# Supplementary material for: A bibliometric and visual analysis of colorectal cancer-diabetes comorbidity
Source: Discov Oncol. 2026 Jan 22;17:313. doi: 10.1007/s12672-026-04447-w (PMC12909731; doi:10.1007/s12672-026-04447-w)
Supplement: Supplementary file 1 — Supplementary Material 1 [file 12672_2026_4447_MOESM1_ESM.docx]

**Supplementary Information
Supplementary Table S1**

|  | **Search Query** | **Results** |
| --- | --- | --- |
| 1 | TI=("Rectal Neoplasm*" OR "Rectum Neoplasm*" OR "Rectal Tumor*" OR "Rectum Cancer*" OR "Rectal Cancer*") | 25856 |
| 2 | AB=("Rectal Neoplasm*" OR "Rectum Neoplasm*" OR "Rectal Tumor*" OR "Rectum Cancer*" OR "Rectal Cancer*") | 23497 |
| 3 | AK=("Rectal Neoplasm*" OR "Rectum Neoplasm*" OR "Rectal Tumor*" OR "Rectum Cancer*" OR "Rectal Cancer*") | 13591 |
| 4 | #1 OR #2 OR #3 | 37028 |
| 5 | AK=("Colonic Neoplasm*" OR "Colon Neoplasm*" OR "Colon Cancer*" OR "Colonic Cancer*" OR "Colon Adenocarcinoma*") | 21198 |
| 6 | AB=("Colonic Neoplasm*" OR "Colon Neoplasm*" OR "Colon Cancer*" OR "Colonic Cancer*" OR "Colon Adenocarcinoma*") | 57225 |
| 7 | TI=("Colonic Neoplasm*" OR "Colon Neoplasm*" OR "Colon Cancer*" OR "Colonic Cancer*" OR "Colon Adenocarcinoma*") | 37112 |
| 8 | #5 OR #6 OR #7 | 79924 |
| 9 | TI=( Diabetes) | 356694 |
| 10 | AB=( Diabetes) | 557036 |
| 11 | AK=( Diabetes) | 229118 |
| 12 | #9 OR #10 OR #11 | 747708 |
| 13 | #4 OR #8 | 113164 |
| 14 | #12 AND #13 | 1208 |
| 15 | LA=(English) | 75896454 |
| 16 | #14 AND #15 | 1170 |
| 17 | DT=(Article) | 50834252 |
| 18 | #16 AND #17 | 905 |

**Supplementary Table S2**

|  | **Title** | **DOI** |  | **Year** | **LCS** | **GCS** |
| --- | --- | --- | --- | --- | --- | --- |
| 1 | History Of Selected Diseases And The Risk Of Colorectal-Cancer | 10.1016/0277-5379(91)90223-Z |  | 1991 | 13 | 79 |
| 2 | Insulin, Insulin-Like Growth Factors And Colon Cancer: A Review Of The Evidence | 10.1093/jn/131.11.3109S |  | 2001 | 33 | 742 |
| 3 | Impact Of Diabetes Mellitus On Outcomes In Patients With Colon Cancer | 10.1200/JCO.2003.07.125 |  | 2003 | 24 | 277 |
| 4 | Diabetes Mellitus As A Predictor Of Cancer Mortality In A Large Cohort Of Us Adults | 10.1093/aje/kwh161 |  | 2004 | 27 | 626 |
| 5 | Diabetes Mellitus And The Risk Of Cancer - Results From A Large-Scale Population-Based Cohort Study In Japan | 10.1001/archinte.166.17.1871 |  | 2006 | 14 | 431 |
| 6 | Effect Of Diabetes Mellitus On The Epidemiology And Outcomes Of Colon Cancer | 10.1385/MO:23:4:515 |  | 2006 | 8 | 27 |
| 7 | A Prospective Study Of Anthropometric And Clinical Measurements Associated With Insulin Resistance Syndrome And Colorectal Cancer In Male Smokers | 10.1093/aje/kwj253 |  | 2006 | 7 | 101 |
| 8 | Serum C-Peptide, Igfbp-1 And Igfbp-2 And Risk Of Colon And Rectal Cancers In The European Prospective Investigation Into Cancer And Nutrition | 10.1002/ijc.22697 |  | 2007 | 4 | 145 |
| 9 | Metabolic Syndrome, Hyperinsulinemia, And Colon Cancer: A Review | 10.1093/ajcn/86.3.836S |  | 2007 | 18 | 402 |
| 10 | Comorbidity In Older Surgical Cancer Patients: Influence On Patient Care And Outcome | 10.1016/j.ejca.2007.06.008 |  | 2007 | 6 | 185 |
| 11 | Systemic Treatment With The Antidiabetic Drug Metformin Selectively Impairs P53-Deficient Tumor Cell Growth | 10.1158/0008-5472.CAN-06-4447 |  | 2007 | 6 | 769 |
| 12 | Diabetes Mellitus Affects Response To Neoadjuvant Chemoradiotherapy In The Management Of Rectal Cancer | 10.1245/s10434-008-9873-6 |  | 2008 | 11 | 47 |
| 13 | The Comparison Of The Risk Factors And Clinical Manifestations Of Proximal And Distal Colorectal Cancer | 10.1007/s10350-007-9083-5 |  | 2008 | 4 | 27 |
| 14 | Type 2 Diabetes Mellitus Associated With Increased Risk For Colorectal Cancer: Evidence From An International Ecological Study And Population-Based Risk Analysis In China | 10.1016/j.puhe.2009.06.019 |  | 2009 | 7 | 34 |
| 15 | Metabolic Syndrome Is Associated With Colorectal Cancer In Men | 10.1016/j.ejca.2010.03.010 |  | 2010 | 4 | 73 |
| 16 | Prospective Study Reveals Associations Between Colorectal Cancer And Type 2 Diabetes Mellitus Or Insulin Use In Men | 10.1053/j.gastro.2010.06.072 |  | 2010 | 8 | 105 |
| 17 | Metabolic Syndrome And Risks Of Colon And Rectal Cancer: The European Prospective Investigation Into Cancer And Nutrition Study | 10.1158/1940-6207.CAPR-11-0218 |  | 2011 | 5 | 107 |
| 18 | Impact Of Diabetes Mellitus And Insulin Use On Survival After Colorectal Cancer Diagnosis: The Cancer Prevention Study-Ii Nutrition Cohort | 10.1200/JCO.2011.38.0303 |  | 2012 | 18 | 89 |
| 19 | Disease-Specific Mortality Among Stage I-Iii Colorectal Cancer Patients With Diabetes: A Large Population-Based Analysis | 10.1007/s00125-012-2555-8 |  | 2012 | 11 | 44 |
| 20 | Diabetes, Metformin Use, And Colon Cancer: A Population-Based Cohort Study In Taiwan | 10.1530/EJE-12-0369 |  | 2012 | 5 | 106 |
| 21 | Diabetes Mellitus And Colorectal Cancer Prognosis: A Meta-Analysis | 10.1097/DCR.0b013e3182a479f9 |  | 2013 | 22 | 139 |
| 22 | Impact Of Diabetes On Oncologic Outcome Of Colorectal Cancer Patients: Colon Vs. Rectal Cancer | 10.1371/journal.pone.0055196 |  | 2013 | 7 | 66 |
| 23 | Type 2 Diabetes Mellitus Is Associated With Increased Mortality In Chinese Patients Receiving Curative Surgery For Colon Cancer | 10.1634/theoncologist.2013-0423 |  | 2014 | 4 | 20 |
| 24 | Type 2 Diabetes, Antidiabetic Medications, And Colorectal Cancer Risk: Two Case-Control Studies From Italy And Spain | 10.3389/fonc.2016.00210 |  | 2016 | 4 | 25 |
| 25 | Relationship Between Metformin Use And Recurrence And Survival In Patients With Resected Stage Iii Colon Cancer Receiving Adjuvant Chemotherapy: Results From North Central Cancer Treatment Group N0147 (Alliance) | 10.1634/theoncologist.2016-0153 |  | 2016 | 6 | 34 |
| 26 | The Relationship Between Diabetes And Colorectal Cancer Prognosis: A Meta-Analysis Based On The Cohort Studies | 10.1371/journal.pone.0176068 |  | 2017 | 11 | 82 |
| 27 | Hyperglycemia Exacerbates Colon Cancer Malignancy Through Hexosamine Biosynthetic Pathway | 10.1038/oncsis.2017.2 |  | 2017 | 4 | 80 |
| 28 | Impact Of Metformin Use And Diabetic Status During Adjuvant Fluoropyrimidine-Oxaliplatin Chemotherapy On The Outcome Of Patients With Resected Colon Cancer: A Tosca Study Subanalysis | 10.1634/theoncologist.2018-0442 |  | 2019 | 4 | 22 |
